# A Simple Algorithm for Finding All *k*-Edge-Connected Components

**DOI:** 10.1371/journal.pone.0136264

**Published:** 2015-09-14

**Authors:** Tianhao Wang, Yong Zhang, Francis Y. L. Chin, Hing-Fung Ting, Yung H. Tsin, Sheung-Hung Poon

**Affiliations:** 1 Research Center for High Performance Computing, Shenzhen Institutes of Advanced Technology, Chinese Academy of Sciences, Shenzhen, China; 2 Software School, Fudan University, Shanghai, China; 3 College of Mathematics and Information Science, Hebei University, Baoding, China; 4 Department of Computer Science, The University of Hong Kong, Hong Kong, China; 5 Hang Seng Management College, Hong Kong, China; 6 School of Computer Science, University of Windsor, Windsor, Canada; 7 School of Computing and Informatics, Institut Teknologi Brunei, Gadong, Brunei Darussalam; University of Connecticut, UNITED STATES

## Abstract

The problem of finding *k*-edge-connected components is a fundamental problem in computer science. Given a graph *G* = (*V*, *E*), the problem is to partition the vertex set *V* into {*V*_1_, *V*_2_,…, *V*_*h*_}, where each *V*_*i*_ is maximized, such that for any two vertices *x* and *y* in *V*_*i*_, there are *k* edge-disjoint paths connecting them. In this paper, we present an algorithm to solve this problem for all *k*. The algorithm preprocesses the input graph to construct an *Auxiliary Graph* to store information concerning edge-connectivity among every vertex pair in *O*(*Fn*) time, where *F* is the time complexity to find the maximum flow between two vertices in graph *G* and *n* = ∣*V*∣. For any value of *k*, the *k*-edge-connected components can then be determined by traversing the auxiliary graph in *O*(*n*) time. The input graph can be a directed or undirected, simple graph or multigraph. Previous works on this problem mainly focus on fixed value of *k*.

## 1 Introduction

Graph connectivity is a fundamental problem in computer science, which has many background applications in the real world. For example, reliability is one of the major concerns in communications networks: if a network is reliable, the network would still work when some nodes or edges fail. Reliability in communication networks can be represented by the connectivity between each pair of nodes. In social networks, computing the closeness among people is a very important problem, which also relates to the connectivity of the networks. There are many other applications which are related to the connectivity of networks, e.g., finding web pages of high commonality in internet searching; finding protein complexes and gene clusters in computational biology, etc.

In theoretical computer science, graph connectivity has been well studied for more than forty years. It has a strong relationship with the problems of maximal network flow and minimal cut.

**Fig 1:**
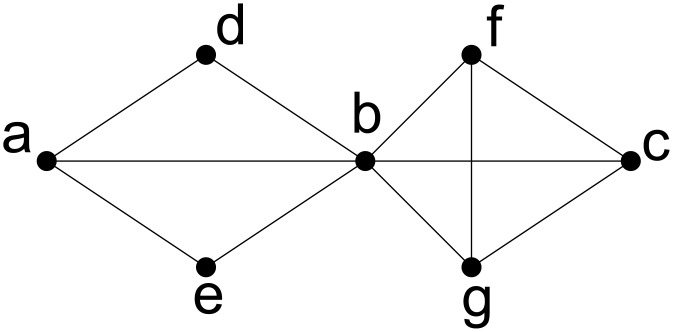
Edge connectivity in undirected graphs.

Given an *undirected graph G* = (*V*, *E*), where *V* is the vertex set and *E* is the edge set, an edge set *E*_*c*_, which is a subset of *E*, is an *edge-cut* of nodes *x*, *y* ∈ *V*, if removing all edges in *E*_*c*_ disconnects *x* and *y* in *G*. We say that *x* and *y* are *k-edge-connected* in *G* if there is no edge-cut disconnecting *x* and *y* with cardinality strictly less than *k*. In other words, there are at least *k* edge disjoint paths connecting *x* and *y*. From the definition, if two vertices *x* and *y* are *i-edge-connected*, they must be *j-edge-connected* for any integer *j* < *i*. If any vertex pair is at least *k*-edge-connected in *G*, we say that graph *G* is *k*-edge-connected. For example, in Fig 1, the entire graph is 2-edge-connected and obviously 1-edge-connected too.

Instead of just determining whether the entire graph *G* is *k*-edge-connected, we are interested in finding subsets of vertices which are *k*-edge-connected in *G*. Note that each vertex is *k*-edge-connected to itself. For example, in social networks, finding a group of people who are strongly connected is more important than computing the connectivity of the entire social network. In an undirected graph *G* = (*V*, *E*), a vertex set *V*′ ⊆ *V* is a *k-edge-connected component* if it is a maximal subset of *V* such that for any two vertices *x*, *y* ∈ *V*′, *x* and *y* are at least *k*-edge-connected in *G*. For example, in Fig 1, {*a*, *b*, *c*, *f*, *g*} is a 3-edge-connected component. It is easy to see that *k*-edge-connectivity is an equivalence relation in *V*. Thus, the set of the *k*-edge-connected components forms a partition of *V*. The 2-edge-connected and 3-edge-connected components for the example in Fig 1 are {*a*, *b*, *c*, *d*, *e*, *f*, *g*} and {*a*, *b*, *c*, *f*, *g*}, {*d*}, {*e*} respectively. The collection of *k*-edge-connected components is a partition of *V* immediately implies that every *k*-edge-connected component is unique and maximal.

For a directed graph *G* = (*V*, *E*), let *u*, *v* ∈ *V*. Let *k*_*uv*_ be the maximum number of edge-disjoint directed paths from *u* to *v*, and *k*_*vu*_ be the maximum number of edge-disjoint directed paths from *v* to *u*. Then, *u* and *v* are *k-edge-connected*, where *k* = min{*k*_*uv*_, *k*_*vu*_}. Notice that the directed paths from *u* to *v* and the directed paths from *v* to *u* may not be edge-disjoint. Moreover, *k*_*uv*_ and *k*_*vu*_ may be different. A *k-edge-connected component* of *G* is a maximal subset *V*′ of *V* such that ∀*u*, *v* ∈ *V*′, *u* and *v* are at least *k*-edge-connected.

**Fig 2:**
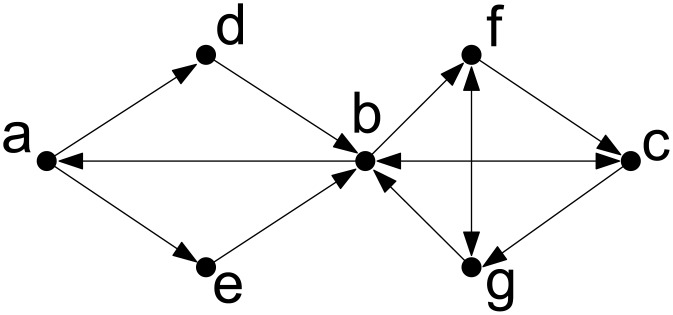
Edge connectivity in directed graphs.

An *edge-cut* of *u* and *v* is an edge set whose removal destroys either all the directed paths from *u* to *v* or all the directed paths from *v* to *u*. A *min-cut* of *u* and *v* is an edge-cut of *u* and *v* with minimum cardinality. If *u* and *v* are *k*-edge-connected, the cardinality of their min-cut is *k*. In Fig 2, all vertices are 1-edge-connected and {*b*, *c*, *f*, *g*} are 2-edge-connected.

**Problem Statement:** Given a graph *G* = (*V*, *E*), find the *k*-edge-connected components of *G* for every *k* ≥ 1.

### Related works

Finding *k*-edge-connected components of undirected graphs has been extensively investigated, especially for some small *k*.

For *k* = 1, this problem is equivalent to finding the connected components of *G*, which can be done in linear time by a single breadth first search or depth first search.

For *k* = 2, based on depth-first search, Tarjan presented an algorithm for finding all 2-edge-connected components in linear time in [1].

For *k* = 3, the first linear time algorithm was given by Galil and Italiano [2]. Their method is to reduce the 3-edge-connectivity problem to the 3-vertex-connectivity problem and then apply Hopcroft and Tarjan’s algorithm for 3-vertex-connectivity to solve the problem. Unfortunately, the algorithm is quite complicated. Many more practical linear-time algorithms [3–6] were given later. All of these algorithms are based on depth-first search. Depth-first search (DFS) is a very useful tool for computing connectivity, which partitions the edges in graph *G* into tree edges and back edges. The tree edges form the DFS-tree while each back edge forms a cycle with a path in the DFS tree. Thus, back edges may be considered one by one to increase the connectivity between vertices.

For general *k*, Matula [7] gave an algorithm to determine the edge-connectivity of *G* = (*V*, *E*) in *O*(∣*V*∣∣*E*∣) time. He also showed that given *k* in advance, testing whether a graph is *k*-edge-connected can be done in *O*(*k*∣*V*∣^2^) time. Nagamochi and Watanabe [3] gave an $O(|V|min(k,|V|,\sqrt{\left|E\right|})|E|)$-time algorithm for finding all *k*-edge-connected components in a direct or undirected graph *G* = (*V*, *E*) given *k* in advance. Using the reduction in [8], i.e., any *k*-edge-connected undirected graph *G* = (*V*, *E*) has a *k*-edge-connected spanning subgraph *G*′ = (*V*, *E*′) with ∣*E*′∣ = *O*(*k*∣*V*∣). They showed that the time complexity can be reduced to *O*(∣*E*∣ + *k* ^2^∣*V*∣^2^) for undirected graphs. This result is also based on an interesting observation: if the cardinality of a cut (*X*, *V* − *X*) is strictly less than *k*, then the edge connectivity of any two vertices *x* and *y* such that *x* ∈ *X* and *y* ∈ *V* − *X* is also strictly less than *k*. In this case, there is no need to consider such vertex pair (*x*, *y*) since *x* and *y* must be in different *k*-edge-connected components. Thus, *O*(∣*V*∣) time for finding min-cut of vertex pairs is sufficient. In database community, k-edge-connectivity problem is also a well studied research topic [9, 10]. Based on a graph decomposition paradigm, Chang et al. [10] gave an *O*(*hl*∣*E*∣) time algorithm, where *h* is the height of the decomposition tree of the graph, and *l* is a small number which is less than ∣*V*∣.

Since the edge connectivity of two vertices *u*, *v* ∈ *V* can be represented by the cardinality of the minimum (*u*, *v*)-cut in *G*, if the minimum (*u*, *v*)-cut can be computed efficiently for all pairs of vertices, the edge connectivity problem could be solved efficiently. The cut tree (a.k.a. Gomory-Hu tree) [11] is a good candidate structure to represent the minimum (*u*, *v*)-cut for all pairs of vertices. A cut tree is a tree *T* = (*V*, *E*_*T*_), where each edge (*u*, *v*) ∈ *E*_*T*_ has a weight which represents the cardinality of the minimum (*u*, *v*)-cut in *G*, and the cardinality of the minimum (*s*, *t*)-cut for any two vertices *s* and *t* ∈ *V* is the minimum edge weight on the path connecting *s* and *t* in the cut tree *T*. To construct the cut tree, there are mainly two methods from Gomory and Hu [11] and Gusfield [12], respectively. Both methods need to compute the minimum cut for some designated pair of vertices. The cut tree can be only used to compute the edge connectivity for undirected graphs. For directed graphs, Schnorr [13] introduced *β* cut-tree to show the edge connectivity between vertices in directed graphs. From the *β* cut-tree, the minimal of the maximal number of edge disjoint paths for any pair of vertices can be found easily. Schnorr [13] constructed the *β* cut-tree by calculating the maximal flow *O*(∣*V*∣ log ∣*V*∣) times, which was improved to 3∣*V*∣ − 3 times by Gusfield and Naor [14].

### Methods and Contributions

Previous works focused on finding the *k*-edge-connected components when *k* is given in advance, especially for some small *k*, e.g., *k* = 2, *k* = 3, or the input graph is an undirected graph. Our algorithm can give answers for all possible values of *k*, and for both directed and undirected, simple graph or multiple graph.

If the capacity of each edge is regarded as one, computing *k*-edge-connected component can be solved by executing an algorithm for max-flow (or min-cut, by the max-flow min-cut theorem [15]). Since the cardinality of the minimum edge-cut separating vertices *a* and *b* is the number of edge-disjoint paths between *a* and *b*, a naive idea is to run an *s-t* min-cut algorithm for each pair of vertices on the graph. If we use an *O*(*n* ^3^) time algorithm proposed by Goldberg and Tarjan [16], we can achieve an *O*(*n* ^5^) time algorithm to get the min-cut of any two vertices. The other method is to use the global min-cut algorithm proposed by Stoer and Wagner [17], which finds the global min-cut in *O*(*mn* + *n* ^2^ log *n*) time. If the min-cut capacity is more than *k*, the graph is a *k*-edge-connected component; otherwise, any two vertices separated by the cut cannot be in the same *k*-edge-connected component. In the worst case, the global min-cut algorithm can be executed *n* − 1 rounds, leading to an *O*(*n* ^2^ *m* + *n* ^3^ log *n*) time algorithm.

In this paper, we give a simple algorithm to find the *k*-edge-connected components for all *k* in a directed or undirected, simple or multiple graph. We use an *s-t* max-flow algorithm as the basic procedure which is executed 2*n* − 2 rounds to construct an auxiliary graph to store information concerning the edge-connectivity between all vertex pairs of the input graph. The time complexity to construct the auxiliary graph is *O*(*Fn*), where *F* is the time required to compute the maximal flow between two vertices in graph *G*, e.g., for the maximal flow algorithm of Ford and Fulkerson [18], *F* = *O*(*fm*), where *f* is the maximal value of all pairs of maximal flows, and for the algorithm by Goldberg and Tarjan [16], *F* = *O*(*n* ^3^). Furthermore, any improvement made on *F* automatically implies improvement on the time complexity of our algorithm.

After the auxiliary graph is constructed, for any value of *k*, the *k*-edge-connected components can then be determined by traversing the auxiliary graph in *O*(*n*) time by a simple scan over the auxiliary graph.

### Outline of this paper

In Section 2, a data structure, called *auxiliary graph*, which could be used to efficiently find all *k*-edge-connected components for all *k* is introduced. In Section 3, the procedure Construction for constructing the auxiliary graph is presented. The correctness proof and the complexity analysis are given in Section 4 and Section 5, respectively. Finally, we give the concluding remark in Section 6.

## 2 Auxiliary Graph

**Fig 3:**
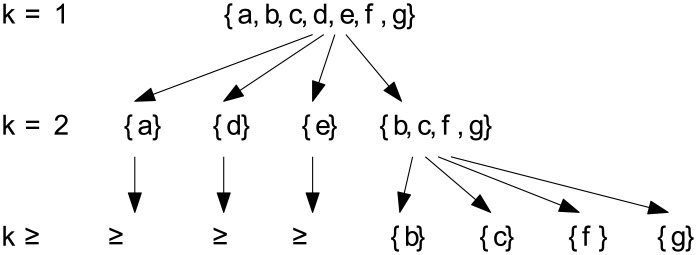
The components for all *k* derived from the graph in Fig 2.

It is well-known that the set of *k*-edge-connected components (*k* ≥ 1) is a partition of *V*. Moreover, each *k*-edge-connected component is the union of a collection of (*k* + 1)-edge-connected components. Fig 3 depicts the *k*-edge-connected components of the graph in Fig 2 for *k* ∈ {1, 2, 3} (note that for *h* > 3, the collection of *h*-edge-connected components is identical to the collection of 3-edge-connected components which is a collection of singletons each of which consists of a distinct vertex in *V*). Consider *k* = 2. The 2-edge-connected component {*b*, *c*, *f*, *g*} is the union of the collection of 3-edge-connected components {*b*}, {*c*}, {*f*}, {*g*}. For the remaining three 2-edge-connected components, each of them is the union of the collection consisting of the component itself. For *k* = 1. There is only one 1-edge-connected component {*a*, *b*, *c*, *d*, *e*, *f*, *g*} which is the union of the collection of 2-edge-connected components {*a*}, {*d*}, {*e*}, {*b*, *c*, *f*, *g*}.

Owing to this hierarchical structure of *k*-edge-connected components, it is possible to store the *k*-edge-connected components, for all *k* ≥ 1, in a compact form in a data structure which we call *auxiliary graph*. The auxiliary graph, henceforth denoted by *A*, is a weighted undirected tree with vertex set *V*. Let *h* be the smallest integers such that the *h*-connected components are singletons. Let *A*_*h*_ be the edgeless spanning forest of *A*. Then the collection of all *h*-edge-connected components of *G* is the collection of vertex sets of the connected components of *A*_*h*_ (each of which consists of a single vertex). Let *A*_*h*−1_ be a spanning forest of *A* obtained from adding the edges of weight *h* − 1 of *A* to *A*_*h*_. Then the collection of all (*h* − 1)-edge-connected components of *G* is the collection of vertex sets of the connected components of *A*_*h*−1_. In general, for *k* < *h*, let *A*_*k*+1_ be a spanning forest of *A* such that the collection of all (*k* + 1)-edge-connected components of *G* is the collection of vertex sets of the connected components of *A*_*k*+1_. Let *A*_*k*_ be a spanning forest of *A* obtained from adding the edges of weight *k* of *A* to *A*_*k*+1_. Then the collection of all *k*-edge-connected components of *G* is the collection of vertex sets of the connected components of *A*_*k*_.

**Fig 4:**
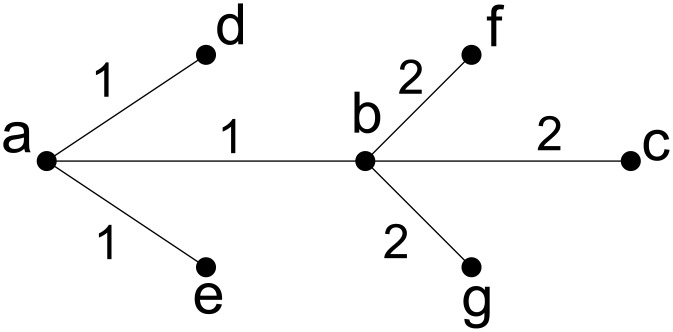
An Auxiliary Graph derived from the graph in Fig 2.

Fig 4 shows an auxiliary graph of the directed graph in Fig 2. The collection of all 3-edge-connected components is represented by the edgeless spanning forest (i.e. {*a*}, {*b*}, {*c*}, {*d*}, {*e*}, {*f*}, {*g*}). The collection of all 2-edge-connected components is represented by the spanning forest induced by the edge set consisting of edges of weight two. The collection of all 1-edge-connected components is represented by the spanning forest induced by the edge set consisting of edges of weight one or two which is the auxiliary graph itself. Notice that in this subgraph, the edges of weight one connect subtrees that represent 2-edge-connected components.

**Auxiliary Graph:** *A* = (*V*, *E*_*A*_) is a weighted undirected tree, which represents the edge connectivity among vertices in *G*. Two vertices *u*, *v* ∈ *V* is *k*-edge-connected in *G* if *k* is the minimum edge weight on the path connecting *u* and *v* in *A* (see Lemma 4). Fig 4 gives an auxiliary graph of the graph in Fig 2. The auxiliary graph *A* is constructed by procedure Construction.

In the next section, we shall explain how to construct the auxiliary graph and how to generate the collection of all *k*-edge-connected components, for any value of *k*.

## 3 Algorithm Description

To determine the weights of the edges in the auxiliary graph, we can use any *s-t* max-flow algorithm, e.g., Ford and Fulkerson algorithm [18].

Since the max-flow algorithms run on directed graphs only, if the input graph *G* is undirected, then each of its edges will be replaced by two directed edges with opposite orientations.

In procedure Construction (see Algorithm 1), on receiving a graph *G* = (*V*, *E*), a vertex *s* (the source) and a set of available vertices *N* (vertices that can be chosen as the sink), the algorithm randomly picks a vertex *t* ∈ *N* − {*s*}, and runs the max-flow algorithm to determine the max-flow from *s* to *t*. If *G* is a directed graph, it would also run the max-flow algorithm to determine the max-flow from *t* to *s* because the two max-flow values can be different. After this step, if *G* is directed, we will obtain two min-cuts, (*S*, *T*) and (*S*′, *T*′). Since the connectivity of *s*, *t* in a directed graph is the minimum of the *s* − *t* max-flow and *t* − *s* max-flow, the smaller of these two max-flows, say *x*, is assigned to edge (*s*, *t*) as the connectivity between *s* and *t*. We also set (*S*, *T*) to the corresponding min-cut (for the case where *G* is undirected, (*S*, *T*) is already the desired min-cut). Then, an edge (*s*, *t*) with weight *x* is added to the auxiliary graph *A*. The procedure then calls itself recursively, first with *S* as the set of available vertices and *s* as the source, and then with *T* as the set of available vertices and *t* as the source. The recursive calls terminate when *S* or *T* is reduced to a single vertex.

**Algorithm 1: Construction**(G(V, E), s, N)

**If** *N* = {s}

**Return**.

 Randomly pick a vertex *t* from *N* − {s}.

 (*x*, *S*, *T*) ≔ s-t max-flow(*G*, *s*, *t*).

 (*x*′, *T*′, *S*′) ≔ s-t max-flow(*G*, *t*, *s*).

**If** *x*′ < *x*

*x* ≔ *x*′, *S* ≔ *S*′, *T* ≔ *T*′

 Add edge (*s*, *t*) with weight *x* to *A*

 Construction(*G*, *s*, *N* ∩ *S*)

 Construction(*G*, *t*, *N* ∩ *T*)

After the auxiliary graph *A* is constructed, for each query *k*, the *k*-edge-connected components can be easily determined as follows: traverse the auxiliary graph *A* and delete all edges with weights less than *k*. Then, each connected component in the resulting graph represents a *k*-edge-connected component in *G*. Since the number of edges in the auxiliary graph *A* is *n* − 1 (see Lemma 5), a search on *A* can be done in *O*(*n*) time, e.g., running the depth-first-search.

## 4 Correctness of The Algorithm

**Lemma 1**. *The connectivity of vertices is transitive. Let C(p,q) denote the connectivity between p and q. Let C(a, b) = x, C(b, c) = y, C(a, c) = z, then*

z≥min(x,y).

*Proof*. Suppose to the contrary that *z* < *min*(*x*, *y*). Let *C* be a min-cut for *a* and *c*. Then ∣*C*∣ = *z* < *min*(*x*, *y*) ⇒ ∣*C*∣ < *x* and ∣*C*∣ < *y*. Since ∣*C*∣ < *x*, *C* is not an edge-cut of *a* and *b*. Therefore, there is a directed path from *a* to *b* and a directed path from *b* to *a* after *C* is removed. Similarly, ∣*C*∣ < *y* implies that there is a directed path from *b* to *c* and a directed path from *c* to *b* after *C* is removed. It follows that there is a directed path from *a* to *c* and a directed path from *c* to *a* after *C* is removed. This contradicts the assumption that *C* is a min-cut for *a* and *c*.

After executing Procedure Construction(*G*, *s*, *t*), the vertex set of *G* is partitioned into subsets *S* and *T* such that *s* ∈ *S* and *t* ∈ *T*.

**Lemma 2**. *For any s′ ∈ S and t′ ∈ T, C(s′,t′) ≤ C(s, t)*.

*Proof*. Let *C*(*x* → *y*) to be the value of the minimal cut between *x* and *y* in graph *G*. Let *x*_1_ = *C*(*s* → *t*) and *x*_2_ = *C*(*t* → *s*). W.l.o.g., *x*_1_ ≤ *x*_2_, thus, the connectivity between *s* and *t* is *x*_1_ and from Procedure Construction(*G*, *s*, *t*), the partition *S* and *T* are computed according to the cut from *s* to *t* such that *s* ∈ *S* and *t* ∈ *T*. For any *s*′ ∈ *S* and *t*′ ∈ *T*, we have *C*(*s*′ → *t*′) ≤ *C*(*s* → *t*), thus, the connectivity between *s*′ and *t*′ satisfies *C*(*s*′, *t*′) = min{*C*(*s*′ → *t*′), *C*(*t*′ → *s*′)} ≤ *C*(*s*′ → *t*′) ≤ *C*(*s* → *t*) = *C*(*s*, *t*).

**Lemma 3**. *Let w(a, b) denote the weight of an edge (a, b) in the auxiliary graph A(V, E_A_). Then*,

$$\forall \left(s,t\right),\left(t,u\right)\in E_{A},C\left(s,u\right)=min\left(w\left(s,t\right),w\left(t,u\right)\right).$$

**Fig 5:**
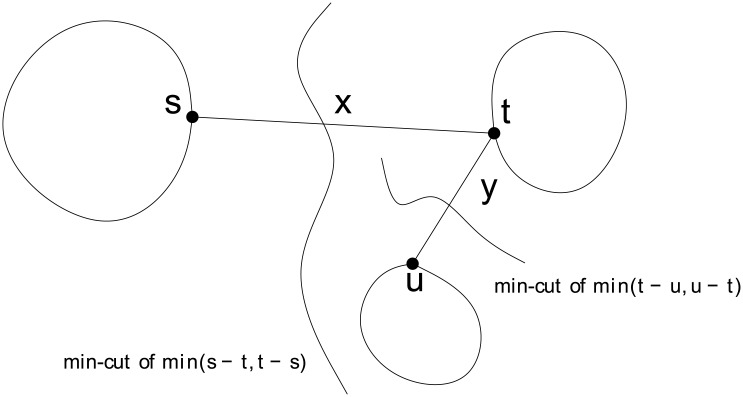
*s* − *u* connectivity is the minimum of *x* and *y*.

*Proof*. By the construction of the auxiliary tree *A*, *w*(*s*, *t*) = *C*(*s*, *t*), for every edge (*s*, *t*). As shown in Fig 5, let *C*(*s*, *t*) = *x*, *C*(*t*, *u*) = *y*, *C*(*s*, *u*) = *z*.

Without loss of generality, assume we determine the value of *x* first. Since *y* has not yet been determined, we must have *s* ∈ *S*; *t*, *u* ∈ *T* (*t*, *u* are on the same side of the cut, while *s* is on the opposite side). From Lemma 2, we have *z* = *C*(*s*, *u*) ≤ *C*(*s*, *t*) = *x*. After *y* is determined, two cases are to be considered.

If *y* ≥ *x*: Since *z* ≤ *x*, then min(*x*, *y*) = *x* ⇒ *z* ≤ min(*x*, *y*). But from Lemma 1, we have *z* ≥ min(*x*, *y*). We thus have *z* = min(*x*, *y*) ⇒ *C*(*s*, *u*) = min{*C*(*s*, *t*), *C*(*t*, *u*)} = min(*w*(*s*, *t*), *w*(*t*, *u*)).

Otherwise, *y* < *x* and we need to prove that *y* ≥ *z*. By Lemma 1, *C*(*t*, *u*) ≥ min{*C*(*t*, *s*), *C*(*s*, *u*)} ⇒ *y* ≥ min{*x*, *z*} ⇒ *y* ≥ *z* as *z* ≤ *x*. It follows that *z* ≤ *y* < *x* which implies that *z* ≤ min{*x*, *y*} = min{*C*(*s*, *t*), *C*(*t*, *u*)} = min{*w*(*s*, *t*), *w*(*t*, *u*)}.

**Lemma 4**. *The auxiliary graph A is a tree and the connectivity between any two vertices in G is given by the minimum weight on the path connecting them in A*.

*Proof*. First, we shall prove that *A* is a tree by induction on ∣*N*∣ (note that *V* = *N*, initially).

When ∣*N*∣ = 1 or 2, *A* is clearly a tree. Suppose *A* is a tree for ∣*N*∣ < *h*(≥ 3).

Consider ∣*N*∣ = *h*. Procedure Construction(*G*, *s*, *N*) is invoked first, resulting in the edge (*s*, *t*) and the min-cut (*S*, *T*). Then Construction(*G*, *s*, *S*) and Construction(*G*, *t*, *T*) are invoked. Since ∣*S*∣,∣*T*∣ < *h*, by the induction hypothesis, the auxiliary graph with vertex set *S* and that with vertex set *T* are trees. The two trees and the edge (*s*, *t*) forms a tree which is the auxiliary graph with the vertex set *N*(= *S*∪*T*).

Next, to prove that the connectivity between any two vertices in *G* is given by the minimum weight on the path connecting them in *A*, we apply induction on the length of the path.

Let *x*, *y* be any two distinct vertices. Since *A* is a tree, there is a distinct path *P* connecting them in *A*. Suppose *P* consists of *h* edges and *h*+1 vertices, such that the length of *P* is *h*, and *P* = (*x* =)*v*_0_ *v*_1_ *v*_2_…*v*_*h*_(= *y*). If *h* = 1, the lemma clearly holds true. If *h* = 2, the lemma follows from Lemma 3.

Suppose the lemma holds true for *h* < *k*(≥ 3). Consider *h* = *k*. Whenever an edge is added to the auxiliary graph *A*, it always connects a node with an isolated vertex in the current configuration of *A*, making the isolated vertex a leaf node of the updated *A*. Thus, when the path *P* = (*x* =)*v*_0_ *v*_1_ *v*_2_…*v*_*h*_(= *y*) is constructed, either *v*_0_ or *v*_*h*_ is the newly added vertex. W.l.o.g., let *v*_*h*_ be the newly added vertex. From the induction hypothesis, *C*(*x*, *v*_*h*−1_) = min{*w*(*v*_*i*_, *v*_*i*+1_)∣1 ≤ *i* < *h* − 1}. Consider the three vertices *x*, *v*_*h*−1_ and *v*_*h*_, similar to the proof of Lemma 3, it is easily verified that *C*(*x*, *v*_*h*_) = min{*C*(*x*, *v*_*h*−1_), *C*(*v*_*h*−1_, *v*_*h*_)}. It follows that *C*(*x*, *v*_*h*_) = min{min{*w*(*v*_*i*_, *v*_*i*+1_)∣1 ≤ *i* < *h* − 1}, *C*(*v*_*h*−1_, *v*_*h*_)} = min{min{*w*(*v*_*i*_, *v*_*i*+1_)∣1 ≤ *i* < *h* − 1}, *w*(*v*_*h*−1_, *v*_*h*_)} = min{*w*(*v*_*i*_, *v*_*i*+1_)∣1 ≤ *i* < *h*}.

**Theorem 1**. *The algorithm returns the correct auxiliary graph*.

*Proof*. Immediate from Lemma 4.

## 5 Complexity Analysis of The Algorithm

Our algorithm works in a preprocessing-query manner. In the preprocessing phase, Procedure Construction is used to construct the auxiliary graph *A*. Then, in the query phase, *A* is used to compute the *k*-edge-connected components for any query *k*.

**Lemma 5**. *There are n − 1 calls of procedure* Construction.

*Proof*. Since each call of procedure Construction adds an edge to *A*, and there are *n* − 1 edges in the finished *A* (Lemma 4 proved *A* is a tree, and thus *A* has *n* − 1 edges), there are *n* − 1 calls of procedure Construction.

**Theorem 2**. *The preprocessing phase takes O(Fn) time and the query phase takes O(n) time per query, where F is the time to compute the maximal flow for two vertices in graph G*.

*Proof*. By Lemma 5, procedure Construction is called *n* − 1 times. In procedure Construction, the basic algorithm for finding the maximal flow and runs in *O*(*F*) time is executed for *n* − 1 times. Therefore, the preprocessing phase takes *O*(*Fn*) time. (Note: if we use the Ford-Fulkerson algorithm [18] to compute the max-flow, the total time complexity is *O*(*fmn*), where *f* is the maximal value of all pair of maximal flows).

Since each query initiates a DFS traversal over *A*, the query time is thus *O*(*m* + *n*). Since the vertex set of *A* is *V* and ∣*V*∣ = *n*, and *m* = ∣*E*_*A*_∣ = *n* − 1 (By Lemma 4, the auxiliary graph is a tree), the query time is *O*(*n*).

## 6 Concluding Remarks

In this paper, an algorithm for finding all *k*-edge-connected components of a graph, for all *k*, is presented. The algorithm performs a preprocessing over the input graph to construct an auxiliary graph which is a tree in *O*(*Fn*) time, where *F* is the time complexity to find the maximum flow in the graph. Clearly, any improvement made on F automatically implies improvement to the time complexity of our algorithm. Every subsequent query asking for the *k*-edge-connected components for any *k*(≥ 1) can be answered in *O*(*n*) time by traversing the auxiliary graph *A*. The input graph can be a directed or undirected, simple or multiple graph.

There are other interesting problems concerning edge-connectivity that are worthwhile to study. For example, a variant of *k*-edge-connected problem has been studied by the database community [10]. In this variant, a subset of vertices *X* is *k*-edge-connected in *G* if for any two vertices in *X*, there are at least *k*-edge disjoint paths within the subgraph *G*(*X*) of *G* induced by *X*. This variant was motivated by finding cores (or cliques) in large scale networks, which is a fundamental database problem. Note that *k*-edge-connectivity remains an equivalence relation under this new definition of *k*-edge-connectivity on induced subgraph, and the vertex partition is always a refinement of the vertex partition of the *k*-edge-connected components.

## References

[1] Tarjan R. E., A note on finding the bridges of a graph, *Information Processing Letters* 2(6): 160–161, 1974 10.1016/0020-0190(74)90003-9

[2] Galil Z. and Italiano G. F., Reducing edge connectivity to vertex connectivity, *SIGACT News* 22, 57–61, 1991 10.1145/122413.122416

[3] Nagamochi H. and Watanabe T., Computing k-edge-connected components of a multigraph, *IEICE Trans.Fundamentals* E76-A(4): 513–517, 1992

[4] Nagamochi H. and Ibaraki T., A linear-time algorithm for computing 3-edge-connected components in a multigraph, *Japan J. Indust. Appl. Math*. 9, 163–180, 1992 10.1007/BF03167564

[5] Tsin Y. H., A Simple 3-Edge-Connected Component Algorithm. *Theory Comput. Syst*. 40(2): 125–142, 2007 10.1007/s00224-005-1269-4

[6] Tsin Y. H., Yet another optimal algorithm for 3-edge-connectivity, *J. Discrete Algorithms* 130–146, 2009 10.1016/j.jda.2008.04.003

[7] D. W. Matula, Determining edge connectivity in *O*(*nm*), *In Proc. of FOCS*, 1987.

[8] Nagamochi H. and Ibaraki T, A linear-time algorithm for finding a sparse k-connected spanning subgraph of a k-connected graph, *Algorithmica* 7(1–6): 583–596, 1992 10.1007/BF01758778

[9] R. Zhou, C. Liu, J. Yu, W. Liang, B. Chen, J. Li, Finding maximal k-edge-connected subgraphs from a large graph. *In Proc. of EDBT 2012*, 480–491.

[10] L. Chang, J. X. Yu, L. Qin, X. Lin, C. Liu, and W. Liang, Efficiently computing k-edge connected components via graph decomposition, *In Proc. of SIGMOD 2013*, 205–216.

[11] Gomory R. E. and Hu T. C., Multi-terminal network flows, *J. Soc. Indust. Appl. Math* 9(4), 551–570, 1961 10.1137/0109047

[12] Gusfield D., Very Simple Methods for all pairs network flow analysis, *SIAM Journal on Computing* 19(1), 143–155, 1990 10.1137/0219009

[13] Schnorr C. P., Bottlenecks and edge connectivity in unsymmetrical networks, *SIAM J. Comput*. 8(2):265–274, 1979 10.1137/0208019

[14] Gusfield D., Naor D., Efficient algorithms for generalized cut-trees, *Networks* 21: 505–520, 1991 10.1002/net.3230210503

[15] Ford L. R. and Fulkerson D. R., Maximal flow through a network, *Canad. J. Math* 8:399–404, 1956 10.4153/CJM-1956-045-5

[16] Goldberg A. V. and Tarjan R. E., A new approach to maximum flow problem, *J. ACM* 35(4), 1988 10.1145/48014.61051

[17] Stoer M. and Wagner F., A simple min-cut algorithm, *J. ACM* 44(4), 1997 10.1145/263867.263872

[18] Ford L. R. and Fulkerson D. R., Flows in Networks, *Princeton University Press* Princeton, New Jersey, 1962.

